# Red blood cell phase separation in symmetric and asymmetric microchannel networks: effect of capillary dilation and inflow velocity

**DOI:** 10.1038/srep36763

**Published:** 2016-11-18

**Authors:** Francesco Clavica, Alexandra Homsy, Laure Jeandupeux, Dominik Obrist

**Affiliations:** 1 ARTORG Center, University of Bern, Bern, Switzerland; 2 Haute Ecole Arc Ingénierie, HES-SO // University of Applied Sciences Western Switzerland, La Chaux-de-Fonds, Switzerland

## Abstract

The non-uniform partitioning or phase separation of red blood cells (RBCs) at a diverging bifurcation of a microvascular network is responsible for RBC heterogeneity within the network. The mechanisms controlling RBC heterogeneity are not yet fully understood and there is a need to improve the basic understanding of the phase separation phenomenon. In this context, *in vitro* experiments can fill the gap between existing *in vivo* and *in silico* models as they provide better controllability than *in vivo* experiments without mathematical idealizations or simplifications inherent to *in silico* models. In this study, we fabricated simple models of symmetric/asymmetric microvascular networks; we provided quantitative data on the RBC velocity, line density and flux in the daughter branches. In general our results confirmed the tendency of RBCs to enter the daughter branch with higher flow rate (Zweifach-Fung effect); in some cases even inversion of the Zweifach-Fung effect was observed. We showed for the first time a reduction of the Zweifach-Fung effect with increasing flow rate. Moreover capillary dilation was shown to cause an increase of RBC line density and RBC residence time within the dilated capillary underlining the possible role of pericytes in regulating the oxygen supply.

The brain requires a constant availability of oxygen and glucose^1^; given its limited energy reserves, normal functioning mainly relies on oxygenated blood provided continuously through the vascular network^2^. Microcirculatory networks in brain as well as in other tissues feature a complex topology with irregularly bifurcating blood vessels^3^,^4^. In such networks, microvascular bifurcations play a significant role in local perfusion because the non-uniform partitioning of red blood cells (RBC) between daughter branches leads to RBC heterogeneity^5^. Non-uniform RBC partitioning has a direct effect on the transport of oxygen and nutrients to living tissues.

At bifurcations RBCs tend to enter the vessel with the higher flow rate (Zweifach-Fung bifurcation effect)^6^ which means that the volume fraction of RBC is reduced (it can even be zero) in the daughter branch with the lower flow rate while it is increased in the daughter branch with higher flow rate^7^. Already more than 40 years ago, Fung noticed the so-called phenomenon of self-regulation of the flow at microvascular bifurcations: RBCs entering predominantly the branch with higher flow cause an increase of the RBC density in that branch which leads to a higher local flow resistance^5^. This results in a reduction of the flow rate difference between the two daughter branches. The basic mechanisms have been extensively investigated in single bifurcations^6^,^8^,^9^,^10^,^11^. In networks of bifurcating (diverging and converging) vessels, RBC dynamics becomes more complex and often counter-intuitive^5^,^12^,^13^,^14^,^15^,^16^,^17^. Computational simulations^8^,^18^ of blood rheology in microcirculatory networks of the rat mesentery have shown that blood flow can undergo spontaneous, self-sustained oscillations in capillaries under steady conditions without any active biological regulation. Recent studies with droplets^13^,^19^,^20^ and RBCs^12^,^21^ suggested that the topology of the network can lead to dynamic changes of the hematocrit (*Ht*) within the network. Computational models of idealized networks^12^ showed that RBC distribution and velocities are irregular and heterogeneous even in uniform networks (uniform vessel length and diameter) although the flow and the RBC density at the inlet were maintained constant.

Next to the complex dynamics of RBC flow which is not yet fully understood, controversial hypotheses exist on the origins of the local increase of oxygen supply during functional hyperemia *in vivo* to compensate the increase of a tissue activity, such as in regional brain activation. There are two prevalent hypotheses: i) the increase is due solely to the action of arteriolar smooth muscle, locally providing higher blood flow rate^22^, or ii) due to a coordinated action of both arteriolar smooth muscle and capillary pericytes (contractile cells which are able to alter the lumen of capillaries)^1^. Recent computational results^21^ showed that capillary dilation/constriction (e.g. due to pericytes) could be a good local regulator of oxygen delivery by varying locally the distribution of RBCs. Moreover very recent findings^23^ indicated that RBCs themselves can act as O_2_ sensors as they can increase their own deformability in response to a decreased O_2_ availability which may directly affect the local RBC distribution.

*In vitro* models are useful to fill the gap between *in vivo* (animal) experiments and *in silico* (computational) models. *In vitro* models do not involve the mathematical modelling (including idealizations and simplifications) required for computational models, at the same time they provide higher controllability than *in vivo* models, e.g. well-defined flow rate and hematocrit at the inflow^14^. *In vitro* models of the microcirculation are particularly useful to i) validate the results of *in silico* models, ii) calibrate techniques used for *in vivo* assessments^24^, iii) investigate local blood flow regulation phenomena, and iv) systematically explore RBC dynamics in different microvascular topologies and under different flow conditions. Many *in vitro* studies so far have focused on RBCs in single capillaries or in single bifurcations with a channel width higher than 20 μm^10^,^25^. Only a few studies, to the best of our knowledge, have focused on the dynamics of RBCs in micro-channels with sizes comparable to blood cells (≤15 μm)arranged as microvascular networks^14^,^17^,^26^,^27^. These studies have provided detailed observations supporting theoretical explanations of the heterogeneous dynamics of RBCs in *in vitro* networks. Strictly theoretical studies of RBC dynamics in microvascular networks are rare^28^. Cybulski and Garstecki^13^,^19^ and Amon *et al*.^20^ were the first to provide a detailed theoretical explanation for the heterogeneous dynamics of droplets in *in vitro* networks. Although these authors used droplets (rather than RBCs) and significantly wider micro-channels, the results are applicable to the microcirculation due to the dynamic similarity of the systems. These studies provided evidence that small changes of cross-section^13^ or length^20^ along specific channels can enable/disable specific patterns of droplets in a network. Nevertheless, it should be kept in mind that the physics of droplets is inherently different from RBCs due to intrinsic mechanical properties of the RBC membrane able to support shear^29^.

In the present study, RBC dynamics in several simple networks of micro-channels are studied *in vitro*. These networks model cerebral microvascular networks where two major fluid dynamic factors may act simultaneously to regulate the partitioning of RBCs: i) change of hydraulic resistance (e.g. dilating/constricting the lumen of capillaries) ii) increase of the overall flow rate (by dilation of feeding arterioles). This could directly affect RBC heterogeneity and local tissue oxygenation in the brain. We hypothesize that these two effects can be observed in our simplified networks, i.e. that the RBC partitioning in our experiments can be affected i) by changing the lumen of the micro-channels and ii) by changing the overall flow rates. To test this hypothesis, we fabricated microdevices embedding three simple microvascular models:

1. A symmetric single mesh (comprising one diverging and one converging bifurcation) which is used as control (symmetric model).
2. An asymmetric single mesh with one branch wider than the other to simulate capillary dilation (dilated model).
3. An asymmetric single mesh model with one branch longer than the other (stretched model).

In all three models, the inflow rate was varied by changing the pressure difference between inlet and outlets. Using particle tracking velocimetry^30^, we provide for the first time *in vitro* quantitative results (i.e. RBC velocity, RBC flux and RBC line density within the network) on the effects of capillary dilation and inflow velocity on phase separation in networks.

## Methods

### RBC suspensions

Fresh venous blood was collected from anaesthetised Swiss white male Landrace pigs. Approval for the animal experiments was obtained from the local Bern University Hospital Animal Experiment Committee. Laboratory and experimental procedures were conducted in accordance with institutional guidelines. Blood (10 ml) was collected and placed in two S-Monovette-2.7-ml blood collection tubes (Sarstedt, Nümbrecht, Germany) which contain Ethylenediaminetetraacetic acid (EDTA) as anticoagulant (concentration: 1.6 mg/ml). The samples were centrifuged at 1800 × g for 5 min to separate cells from plasma^14^. RBCs (approximately 0.5 ml) were collected from the bottom of the centrifuged blood after discarding the supernatant and were re-suspended in a solution of Bovine Serum Albumin (BSA) (Sigma-Aldrich, St Louis, MO, USA) dissolved at 1% in phosphate-buffered saline (Sigma-Aldrich, St Louis, MO, USA). After washing (by gently mixing for 10 minutes) the samples were centrifuged at 2500 × g for 5 minutes. We used this additional washing in BSA solution to reduce echinocytosis as suggested in^31^. All experiments were completed within 6 hours after the blood collection. In order to avoid RBC sedimentation, the final suspending medium was obtained by mixing 65% of GASP buffer (PBS with 5.5 mM glucose and 4% Bovine Serum Albumin) with 35% of stock solution (90% Optiprep (Sigma-Aldrich, St Louis, MO, USA) +10% GASP 10 times concentrated) as suggested by^10^. In this last step, the final suspending medium reached a density similar to RBCs (i.e. 1.09 to 1.11 g/ml). The measured density (ρ_s_) and dynamic viscosity (μ_s_) of the final suspending medium were 1090 kg/m^3^ and 1.96·10^−3^ Pa·s at T = 20 °C, respectively. This is very close to the corresponding viscosity values of plasma from normal subjects (i.e. 1.7–1.92·10^−3 ^Pa·s)^32^. Density ρ_s_ and viscosity μ_s_ were measured respectively with a scale and with a Ubbelohde 501-10 viscosimeter (SI Analytics, Mainz, Germany). For the experiments the RBCs were diluted to a hematocrit of 10%. No leukocytes or platelets were detectable in the final RBC suspension.

### Microdevice fabrication

**Figure 1:**
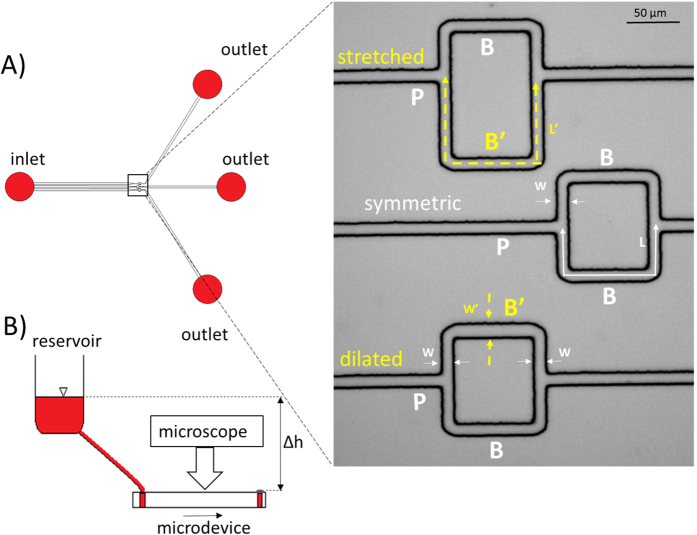
Experimental setup. (**A**) Schematic of the microfluidic device and microscope image (inset, 10× magnification): three different networks were included in the same microdevice, P denotes the parent vessel, B and B’ the daughter branches. Branches denoted as B indicate daughter branches characterized by a total length L = 169 ± 3.2 μm and width W = 10.28 ± 0.3 μm (N = 5). i) Symmetric model (inset, middle) has identical daughter branches B and B’ ii) Dilated model (inset, lower) has one daughter branch B and one modified branch B’ which has same length as B but different width W’ = 13.0 ± 0.2 μm > W (in the horizontal segment) iii) Stretched model (inset, upper) has one daughter branch B and one modified branch B’ with the same width as B but with different length L’ = 253.5 ± 3.1 μm > L (dashed arrows). All models had a common inlet and separate outlets. The average channel height was H = 8.1 ± 0.4 μm in all channels. The parent vessel (P) in all the models had a length of 200 μm and 10.28 ± 0.3 μm width. (**B**) Schematic of the experimental setup: a large diameter reservoir was connected to the inlet of the microdevice. The driving pressure was regulated by changing the height difference (Δh) between the fluid level in the reservoir and the outlets. The arrow indicates the flow direction.

The microfluidic devices were fabricated by molding polydimethylsiloxane (PDMS) on a master which was micro-fabricated in the cleanroom using conventional lithography^14^,^33^. CAD of the microdevice architecture were designed using DraftSight (Dassault Systèmes, Vélizy-Villacoublay, France) and transferred on a chrome photomask (JD Photodata, Hitchin, UK). The master was made from a silicon wafer (Prolog Semicor LTD, Kiev, Ukraine), coated with a 8 μm layer of SU-8 negative photoresist (GM1060 from Gersteltec Sarl., Pully, Switzerland) and then patterned by exposure to UV light (Suss MicroTec Lithography GmbH, Garching, Germany) through the photomask. Liquid PDMS with a curing agent/monomer ratio of 1:10 w/w (Sylgard 184, Dow Corning, Midland, MI, USA) respectively, was poured over the master and degassed in a vacuum chamber for approximately 15 min. It was then cured overnight at 60 °C. Access holes for the inlet and the outlet ports were created using biopsy punches (1.5 mm inner diameter). The PDMS cast was then bonded to a flat PDMS surface using oxygen plasma-activated bonding (Harrick Plasma, Ithaca, NY, USA). The final design of the microdevice is shown in Fig. 1. The resulting three units (symmetric, dilated and stretched model) had a single common inlet and separate outlets, similar to the devices used in ref. 14. The bifurcations were designed as T-junctions. Channel length, width and height were measured using an Axioplan microscope (Carl Zeiss AG, Jena, Germany). The measured values are indicated in Fig. 1 (caption). To assess the influence of the bifurcation geometry on the results, corresponding microdevices with 120° bifurcations were fabricated as well (hexagonal networks, supplementary Figure S1). Results for these junctions are reported in the supplementary material. They did not differ substantially from the results for the T-junctions.

### Experimental protocol

The microdevices were prefilled with GASP buffer before the experiment. The presence of BSA in the GASP buffer prevents the adhesion of blood cells to the PDMS surface^34^. In order to remove the air contained in the microdevices during the filling process, one drop of GASP buffer was added to each inlet/outlet; the microdevices were then placed in a vacuum chamber for approximately 15 minutes. After the vacuum chamber, the complete degassing of the micro-channels was verified by microscope inspection. A reservoir was mounted on a vertical linear-motion stage and connected via plastic tubing to the inlet of the microfluidic devices. Three drops of the suspending medium were always left on the three outlets, carefully preventing them from drying. The hydrostatic pressure difference between the fluid level in the reservoir and the outlets was used as driving pressure of the device. Three different perfusion pressures were used for the experiments: ΔP_1_ = 0.5 cmH_2_O, ΔP_2_ = 1 cmH_2_O, ΔP_3_ = 2 cmH_2_O. Only for the stretched model an additional experiment was conducted for ΔP_4_ = 3 cmH_2_O. The experiments were carried out according to the following sequence: ΔP_3,_ ΔP_1_, ΔP_2_ to avoid potential artifacts due to continuously increasing flow rates. The pressure remained constant throughout the course of each experiment because the change of the fluid level in the reservoir was negligible. The microdevices were mounted on a stage of a microscope (VWR, Radnor, USA) to study the RBC dynamics. For each experiment, videos of 40 s were recorded using a high speed camera (JAI RM-6740 CL, JAI, Denmark), with 640 × 480 pixels and a 25× magnification objective at 100 frames per second.

### Channel resistance

The hydraulic resistance (R) in the channels with rectangular cross-section was calculated according to^35^,


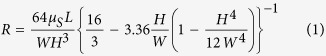


where μ_s_ is the dynamic viscosity of the suspending fluid, L is the length of the channel, W and H are the channel width and height, respectively. The nominal resistances of the two branches of the networks were determined by applying this formula segment by segment.

### Particle tracking velocimetry

The video recordings were processed using a custom-written Matlab (Mathworks, Natick, MA, USA) program to remove the image background and adjust the color table such that the moving RBCs appear as bright white particles on a black background. To this end, an initial frame showing the microdevices without particles was subtracted from each frame. The resulting frames were then imported into the program PTVlab (Mathworks, Natick, MA, USA), an open source software for particle tracking velocimetry^30^. Details on the parameter settings for PTVlab can be found in the supplementary data (Table S1). The algorithm, for each frame, provides the position and velocity vector of each RBC identified in the region of interest (ROI). The PTVlab results were further processed with Matlab to derive the following three signals for each frame in the given ROI: i) average RBC velocity (unit: mm/s): the arithmetic mean of all RBC velocities in the ROI, ii) RBC line density or linear density (unit: RBCs/mm)^36^: the number of RBCs in the ROI of a given frame divided by the total length of the channel segments included in the ROI, iii) average RBCs flux (unit: RBCs/s): resulting from the average RBC velocity multiplied by the RBC line density. In the present study, separate ROIs were defined for each branch of the networks in order to obtain separate measurements. Video frames, in which no RBCs were detected in the ROI of at least one branch, were discarded for the analysis because no velocity could be detected. These discarded frames accounted in general for less than 6% of the total frames. Visual inspection of these frames confirmed that RBCs were actually present in the ROI but that the particle identification algorithm was unable to detect them. The resulting time series were smoothed with a second-order Savitzky-Golay filter Matlab (Mathworks, Natick, MA, USA) using windows of 200 points corresponding to intervals of 2 s.

The line-scan method^36^ was used to validate the PTVlab algorithm, i.e. line scans were sampled from a vessel and were stacked in a temporal sequence. The RBCs were identified from the RBC line traces in these stacks and their velocity was determined from the slope of these lines (more details can be found in ref. 37).

The Wilcoxon rank sum test (ranksum) was performed using the Statistics and Machine Learning toolbox of Matlab (Mathworks, Natick, MA, USA) to compare the velocity, line density and flux at different perfusion pressures (i.e. each ratio with the corresponding ratio for the lowest perfusion pressure). A p-value of less than 0.05 was considered significant.

## Results

**Figure 2:**
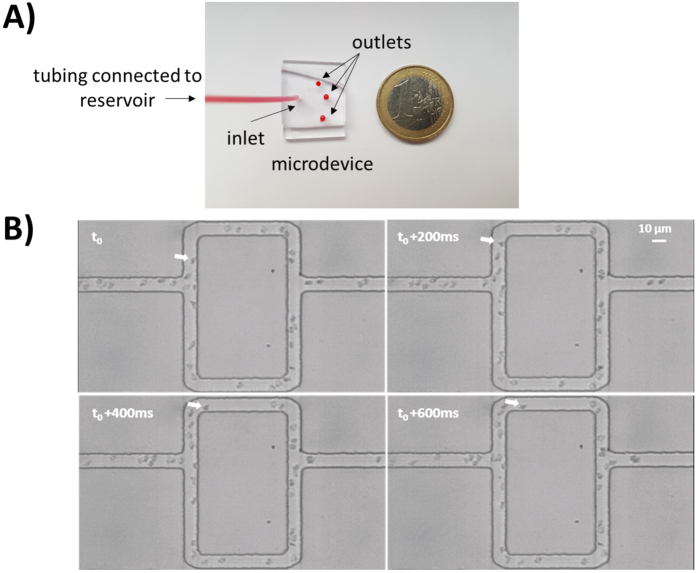
Photos of microdevice (**A**) and video frame sequence of RBCs advancing through microchannels (**B**). The inlet of the microdevice was connected to a reservoir and three droplets were left at the outlet (**A**). The white arrow in panel B shows the same RBC moving in the frame sequence (Δt = 200 ms).

In the following, we will first show the results in a symmetric model which validates our experimental setup and sets a baseline for the experiments with asymmetric networks. Second, we present the effects of local capillary dilation which are an *in vitro* confirmation of earlier findings on the possible role of pericytes that were so far only shown *in silico*^21^. Finally, we present results for the stretched model which demonstrate that the Zweifach-Fung effect depends on the inflow rate. Implications of this new result are presented in the discussion. In Fig. 2 pictures of the microdevice and RBCs advancing in the micro-channels are shown.

### Method validation

The difference between RBC velocity in the parent vessel of the symmetric hexagonal model (at perfusion pressure ΔP_1_) determined by the PTVlab algorithm and the velocity determined by the line-scan method was less than 4% (i.e. 0.32 ± 0.03 mm/s with the PTVlab algorithm, 0.33 ± 0.02 mm/s with the line-scan method). The average RBC line density was 29.9 ± 14.9 RBC/mm with the PTV algorithm and 29.5 ± 12.52 RBC/mm with the line-scan method.

The PTVlab analysis is missing RBCs if their speed is too high leading to large displacements between two consecutive frames. To determine a robust working range for PTVlab, we artificially reduced the frame rate by removing frames from the recordings. This allowed us to identify the RBC velocity limit for the PTVlab algorithm. For our frame rate (100 fps) and magnification (25×) we found that RBC velocities, line densities and fluxes determined with PTVlab were accurate in the range 0 to 0.6 mm/s (velocity difference between PTVlab and line-scan method less than 5%). The range could be increased by increasing the frame rate (e.g. at 200 fps the range extends to 0–1.2 mm/s).

**Figure 3:**
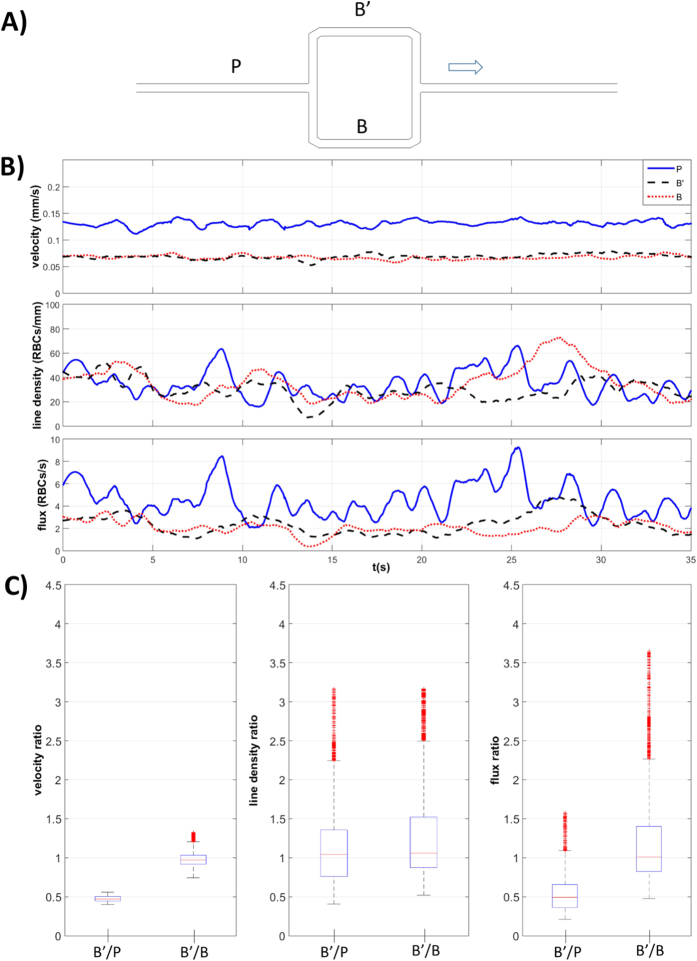
Phase separation results for the symmetric model. (**A**) Schematic of the symmetric model: the two branches B’ and B are identical. The arrow indicates the direction of flow. (**B**) RBC velocity, line density and flux determined using the PTV algorithm, at perfusion pressure ΔP_2_ = 1 cmH_2_O, for the parent vessel (continuous line) and for the two daughter branches (dashed lines) and (**C**) boxplots of the respective ratios: B’/P and B’/B.

In Fig. 3B, RBC velocity, line density and RBC flux are plotted against time for the parent vessel (P) and the two branches (B’ and B) of the symmetric model (Fig. 3A). While the line density is approximately the same for all vessels, RBC velocity and flux in the two daughter branches are half the respective values in the parent vessel, in accordance with mass conservation and symmetric RBC partitioning. This observation is emphasized in Fig. 3C which illustrates the statistics for the ratios between these values for upper branch and the parent vessel (B’/P) and between the two branches (B’/B).

### Asymmetric networks

The hydraulic resistances of the two daughter branches were calculated using Eq. 1 for each model. Table 1 shows the respective ratios of the resistances of the two branches.

These results suggest that a steady perfusion with a homogeneous fluid (e.g. without RBCs, *Ht* = 0%) leads to a 20% higher flow rate in the dilated branch B’ and 33% less flow in the stretched branch B’ of the respective network models, whereas we expect the same flow rate in both branches of the symmetric model. If such a configuration occurs also with an RBC suspension (*Ht* > 0%), we call this a *no-Zweifach-Fung* (NZF) configuration. It corresponds to a situation where the RBC partition is proportional to the respective flow rates in the two daughter branches which leads to the same hematocrit in the daughter branches. For a suspension with a sufficiently high hematocrit, the self-regulation mechanism based on the Zweifach-Fung effect would ideally lead to equal resistance/flow in the two branches also for the asymmetric models. We define this situation as *perfectly self-regulated* (PSR)

For our dilute solution of RBCs (*Ht* ≈ 10%) it was unlikely that we would obtain a PSR situation. Therefore, we expected the RBC velocity ratios in the asymmetric models to be bounded by the two limiting cases: NZF and PSR.

### Effects of capillary dilation

**Figure 4:**
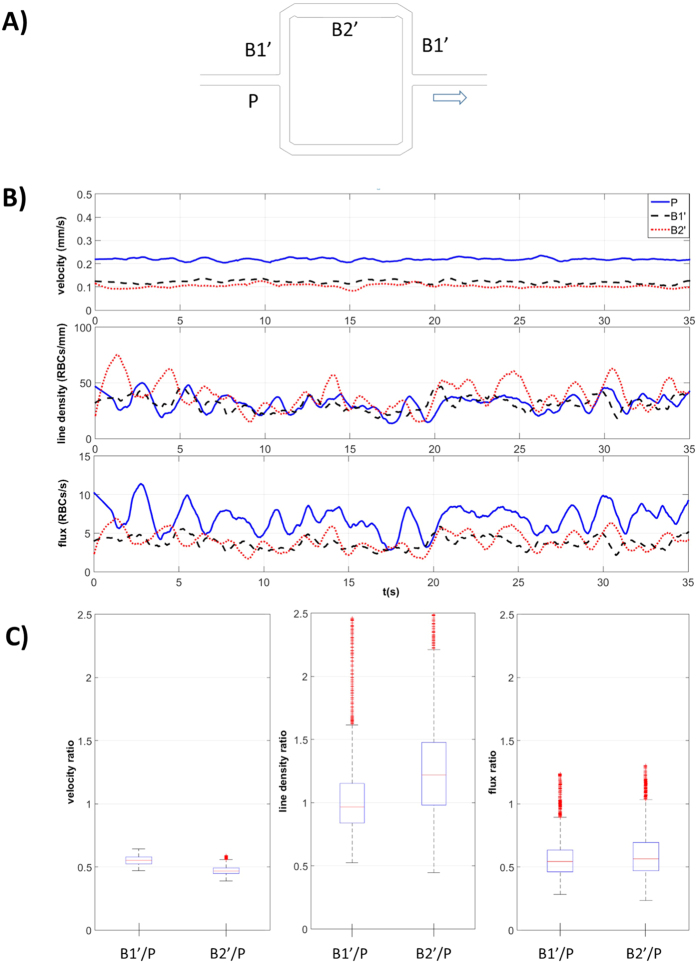
Effects of dilation on phase separation in dilated model. (**A**) Dilated model: schematic of the asymmetric network with the dilated segment B2′ in branch B’. The B1′ segments are not dilated (undilated segments). The arrow indicates the direction of flow. (**B**) RBC velocity, line density and flux determined using the PTV algorithm, at perfusion pressure ΔP_1_ = 0.5 cmH_2_O, for the parent vessel (P, continuous line) and for the two segments of branch B’: dashed lines for the undilated segment B1′ and dotted lines for the dilated segment B2′. (**C**) boxplots of the respective values of B1′ and B2′ normalized by P.

The effects of local capillary dilation on RBC flow are shown in Fig. 4, where we present separately the results for different segments of the partially dilated branch B’ normalized by the values in the parent vessel P: i) undilated segment B1′/P and ii) dilated segment B2′/P.

Local capillary dilation leads to an asymmetric RBC partition with a higher RBC flux toward the partially dilated branch B’ such that the respective velocity and flux ratios in the undilated segment (B1′/P) are higher than 0.5 (Fig. 4C). In accordance with mass conservation, the velocity in the dilated B2′ segment was lower than in B1′ while the RBC flux remained the same. The resulting RBC line density was higher in B2′ than in B1′ and in P (Fig. 4B,C).

### Effects of inflow velocity

**Figure 5:**
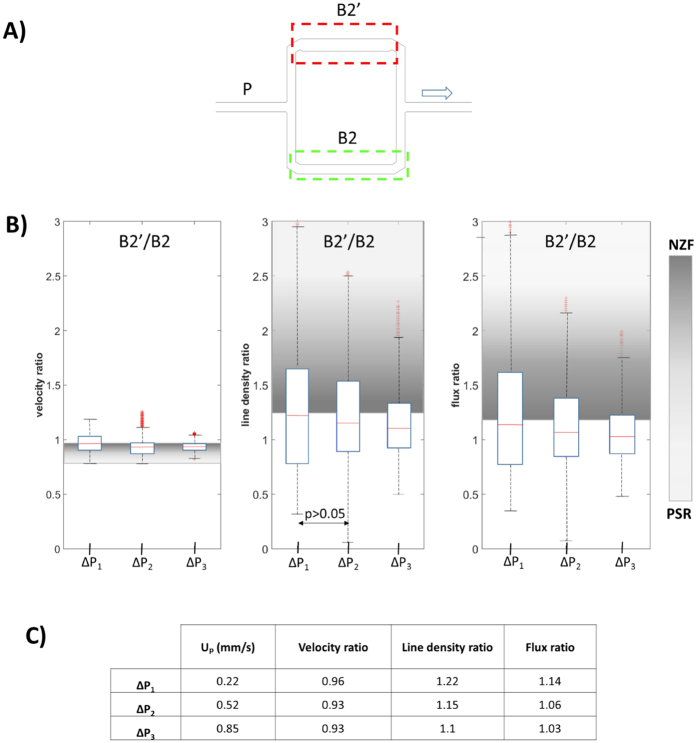
Effects of inflow rate on phase separation in dilated model. (**A**) Dilated model: schematic of the asymmetric network with the dilated segment B2′ in branch B’. The arrow indicates the direction of flow. Red and green dashed rectangles indicate the two regions of interest (ROI). (**B**) Boxplots of the ratios of RBC velocity, line density and flux are of segments B2′ and B2 for different perfusion pressures ΔP_i_. The shaded areas indicate the range between the *no-Zweifach-Fung* condition (dark grey, NZF) and *perfectly self-regulated* situation (light gray, PSR). (**C**) Table of median values of the ratios shown in panel B as function of ΔP. U_p_ indicates the average velocity estimated for the parent vessel.

**Figure 6:**
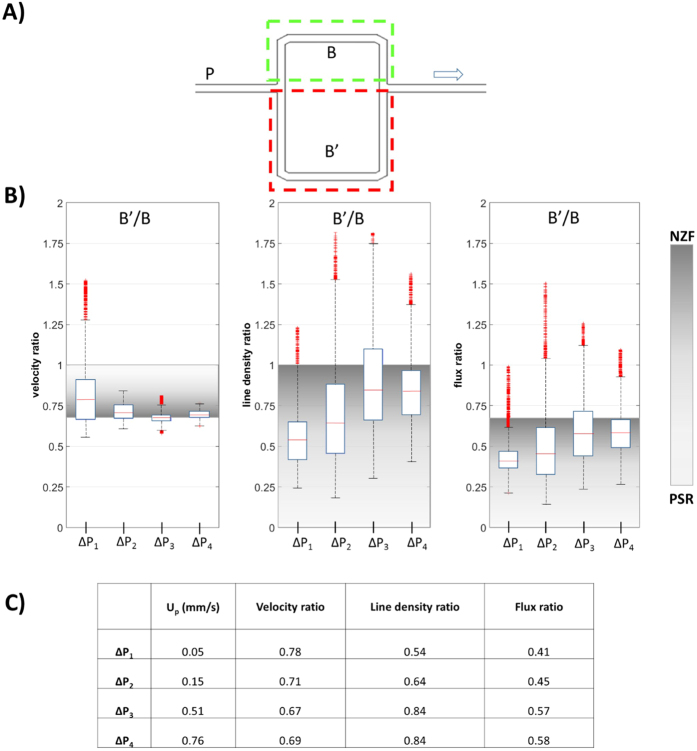
Effects of inflow rate on phase separation in stretched model. (**A**) Schematic of the stretched model with branch B’ (ROI: red dashed rectangle) longer than branch B (ROI: green dashed rectangle). The arrow indicates the direction of flow. (**B**) Boxplots of the rations of RBC velocity, line density and flux of branches B’ and branch B for different perfusion pressures ΔP_i_. The shaded areas indicate the range between the *no-Zweifach-Fung* condition (dark grey, NZF) and *perfectly self-regulated* situation (light gray, PSR). (**C**) Table of median values of the ratios shown in panel B as function of ΔP. U_p_ indicates the average velocity estimated for the parent vessel.

The effect of inflow velocity on RBC flow was investigated in the stretched model as well as in the dilated model. To illustrate the phase separation, we report ratios of the RBC velocities, line densities and fluxes between the modified (dilated or stretched) branch B’ and the normal branch B (Figs 5 and 6 and supplementary Figure S2). In these figures we indicate the range between the two limiting cases (NZF and PSR) with a grey shading passing from dark for the NZF limit to light grey for the PSR situation. At NZF the fluid behaves like a homogeneous Newtonian fluid, such that the flow and flux ratios correspond to the hydraulic resistance ratio (i.e. 1.20 in Fig. 5 and 0.67 in Fig. 6). The velocity ratio is the same as the flow ratio for the stretched model while for the dilated model, due to the wider channel, the NZF velocity ratio B2′/B2 is calculated as: 1.20 × 10.28 μm/13 μm = 0.95. The NZF line density ratio is 1 for the stretched model. For the dilated model, it is calculated as w’/w = 13 μm/10.28 μm = 1.265 (since the cross-section is wider, a given volumetric concentration results in a higher line density). At PSR both branches have the same flow rate by definition. Therefore, the PSR velocity ratio corresponds to w’/w = 10.28 μm/13 μm = 0.79 for the dilated model and it is 1 for the symmetric and stretched models. As mentioned earlier, the number of available RBCs is too low to fully compensate for the difference in nominal resistance and PSR cannot be reached in practice in our experiments. All available RBCs would go to the branch with lower nominal resistance and there would be no RBCs in the branch with higher nominal resistance. Therefore, we set the PSR line density and flux ratios to infinity for Fig. 5 and to zero for Fig. 6.

The p-value for the Wilcoxon ranksum test was found to be less than 0.05 for all data sets. Only the test for the density ratios at ΔP_1_ and ΔP_2_ in the dilated model provided a p-value larger than 0.05 (Fig. 5).

In the stretched model (Fig. 6), the RBC velocity in the stretched branch B’ was lower and (in accordance with the Zweifach-Fung effect) branch B’ had a lower RBC flux, for all the perfusion pressures (ratio B’/B below unity). However, we could observe a significant reduction of the Zweifach-Fung effect with increasing inflow velocity such that the RBC flux ratio between B’ and B approached the NZF condition as the perfusion pressure was increased from ΔP_1_ to ΔP_4_: the RBC flux ratio B’/B increased by 41% from 0.41 to 0.58 (Fig. 6C). At the same time, the velocity ratio B’/B decreased by 12% from 0.78 to 0.69 approaching the limiting hydraulic resistance ratio of 0.67 (Table 1) which corresponds to the NZF condition. This resulted in an increase of the line density ratio B’/B by 55% from 0.54 to 0.84.

The same behavior was also observed in the stretched model with hexagonal design (Supplementary Figure S2)which had a hydraulic resistance ratio B/B’ = 0.81. For this network configuration, the resulting line density ratio B’/B became even greater than unity at ΔP_3_, exceeding the NZF limit. Likewise the RBC flux ratio exceeded the NZF limit although it remained below unity indicating that even in this situation more RBCs entered the higher flow rate branch B per time unit. This phenomenon where the RBC flux ratio exceeds the velocity ratio can be interpreted as an inversion of the Zweifach-Fung effect (as it was also shown by Shen *et al*.^17^).

In the dilated model (Fig. 5), we also observed an inversion of the Zweifach-Fung effect as the flux and the line density ratios are below the threshold NZF value. This effect increases with higher perfusion pressures. This can probably be attributed to the lower difference between the nominal hydraulic resistances of the two branches as we will discuss in the following.

## Discussion

We used microvascular PDMS models to investigate the effects of asymmetric network configurations on blood flow and RBC distribution. The hematocrit (*Ht* ≈ 10%) was chosen within the normal physiologic range in the microcirculation (reported to be in the range of 4–20% i.e. 10% to 48% of the systemic hematocrit refs 38,39). In literature some cortical capillaries at resting conditions were also reported to be without RBCs (Ht = 0%)^40^.

Porcine RBCs have diameters in the range of 4 to 8 μm^41^ which is consistent with the RBCs shown in Fig. 2B. We observed a drastic reduction of the hematocrit when the suspension passed from the reservoir into the microdevice which is in line with other observations^11^. This could be explained by the Fåhræus effect^42^,^43^ and by residual particle sedimentation in the reservoir (despite the use of Optiprep). Considering a line density of 50 RBC/mm (Figs 3 and 4) we calculated our tube hematocrit to be in the range of 3–5%.

The RBC velocities in our experiments were within the typical range (0.3–3.2 mm/s) reported for blood flow in capillaries^36^,^40^ although it has to be kept in mind that the RBC velocity is somewhat higher than the bulk velocity of blood due to the Fåhræus effect^42^,^43^.

There are conflicting opinions on whether RBCs prefer to enter the daughter vessel with higher flow rate^6^,^27^,^44^ or with higher bulk velocity^12^,^21^. In the present study, we circumvent this problem by working with models with equal cross-sections in the daughter branches just after the bifurcation. A symmetric network was used as control and to validate the method. Some of our results confirmed the general behavior due to the Zweifach-Fung effect (Fig. 6). In our experiments, we found characteristic blood flow oscillations in the range 0–0.6 Hz (Figs 3B and 4B) due to the non-linear rheological blood properties, in line with the results of ref. 14. These oscillations are not related to cardiac pulsation which is not modelled here and which is considered negligible at the level of microcirculation due to attenuation by arterial compliance^45^. The presence of self-sustained oscillations in the absence of biological control were shown in microcirculation computationally^46^ even in simple geometries^28^ such as the one considered here. The Fahraeus–Lindqvist effect and Zweifach-Fung effect were considered sufficient^28^ for generating these capillary flow oscillations without any biological control mechanisms.

In this study, two principal phenomena were shown for the first time *in vitro* which may be both important during hyperemia: i) the effects of local capillary dilation (potentially associated to the role of pericytes in blood flow regulation) and ii) the influence of the inflow rate on the Zweifach-Fung effect and thus the RBC heterogeneity (defined as non-uniform distribution of RBCs in a capillary network).

First, we showed with the dilated model that local capillary dilation leads to an increase of RBC line density in the dilated segment which allows for a higher oxygen supply to the local tissue volume. This finding is in line with^21^ who suggested this mechanism based on computational results. Our *in vitro* results showed that an increase of the capillary width (from 10.28 to 13 μm) for half of the length of the daughter branch B’, produced an increase of RBC line density in the dilated segment up to 22% (Fig. 5). Moreover, because of the higher cross sectional area, the dilated segment B2′ showed lower RBC velocity than in the other vessel segments. This could be particularly favorable for oxygen exchange due to higher RBC line density combined with longer residence time. Our results may confirm the potential role of pericytes in microcirculation, especially in the microcirculation of the brain and the retina which show the highest density of pericytes in the body^47^.

The second main result concerned the effects of the inflow velocity on the Zweifach-Fung effect. Experimental data demonstrated a reduction of the Zweifach-Fung effect (and in some cases even an inversion) and therefore a reduction of RBC heterogeneity with increasing the perfusion pressure and inflow velocity. This is in line with observations *in vivo* (in frog’s muscle microcirculation) by Ellis *et al*.^48^ who also noticed a decrease of heterogeneity with flow rate.

In our experiments the width of the channels was bigger than the size of an RBC such that cell margination may occur, i.e. RBCs could be concentrated closer to the channel walls. For this reason, two competing phenomena affect the RBC partitioning at our diverging bifurcations: i) RBCs enter the daughter vessel with higher flow rate (Zweifach-Fung effect), and ii) RBCs follow the streamlines. This situation has been studied computationally by^9^ who showed that (in a locally symmetric bifurcation) RBCs close to the centerline adhere to the Zweifach-Fung effect whereas RBCs closer to the walls (cell margination) rather follow the local streamlines.A conclusive explanation of the partitioning phenomenon is not trivial as, at low hematocrit, the dynamics of single RBCs (like tank-treading or tumbling motion) may have a strong effect on RBC partition^17^; effects on the RBC partition (at low hematocrit) due to cell interactions, initial positions of cells and membrane deformability were reported *in silico* by ref. 16. In our experiments, we have observed tumbling motion predominantly for lower flow velocities. However, a detailed analysis was not possible due to limited image quality. For higher velocities, RBC dynamics might have transitioned to a tank-treading motion where RBC partitioning might have been affected by the angle of inclination of the RBC (with respect to the flow direction) which was found to be function of the shear rate^46^. This rheological behaviour is observed in suspensions of capsules (like RBCs) and not in suspensions of droplets^49^.

Possible inertial effects on our results were excluded as Reynolds number in the parent vessels (calculated with the hydraulic diameter Dh = 4A/P, where A is the channel cross sectional area and P is the perimeter) for all the experiments was below unity (2.5·10^−4^ < Re < 3.8·10^−3^). Likewise, the Stokes number for RBCs was negligibly small. Since increase of the average haematocrit in the parent vessel was reported^44^ to cause a reduction in the phase separation phenomenon, we tested possible effects of changes of haematocrit on our results. To this end, we compared the line density of the parent vessel of the stretched model at the different perfusion pressures but, although a small decrease of the line density from ΔP_1_ to ΔP_2_, the line density did not change significantly (Figure S3).

There are two phenomena which could provide an explanation for the RBC partitioning observed in our experiments: first, a gradual depletion of the central region of the parent vessel will affect RBC partitioning as suggested by Shen *et al*.^17^. In general, such a cell margination leads to an increasingly equal RBC partitioning, i.e. the RBC flux ratio will tend toward unity with increasing cell margination. If the flow rates of the two daughter branches are only slightly different (e.g. as in the dilated model) this effect will lead to a nearly equal RBC partitioning which can be interpreted as an inversion of the ZF effect (as we have observed in Fig. 5).

Second, we speculate that the central depletion increases with flow rate. This will enhance the effect described above, such that higher perfusion pressures will lead to RBC flux ratios increasingly tending toward unity (as seen in Figs 5 and 6 and S2).

### Limitations

A central limitation of the used microfluidic devices is that they are unable to replicate the circular cross section of capillaries due to fabrication issues occurring for channel sizes below 30–40 μm. Therefore, rectangular cross-sections are typically used instead^26^,^27^. Rectangular cross-sections can affect the local fluid dynamics because RBCs usually do not fully plug such micro-channels and plasma is expected to flow past the RBC in the four corner regions^50^. This prevents the pressure build-up which eventually can put a stuck RBC in motion. To mitigate the effects of a rectangular cross-section, we used channels size in the range of 8 to 13 μm (i.e. slightly bigger than the cell size) such that RBCs are unlikely to get stuck. Furthermore, the endothelial surface layer (ESL i.e. macromolecular layer lining the endothelial surface) was found to play an important role on blood flow resistance and its dependence to the flow rates^51^,^52^,^53^. In our microvascular models we were not able to model the ESL, however some promising results have been recently shown using single brush-coated micro-channels as *in vitro* ESL model^54^.

## Conclusions

In the present study we developed simple models of cerebral microvascular networks to investigate the role of two main factors which, according to literature, are considered relevant during hyperemia: i) capillary pericytes (contractile cells which are able to alter the lumen of capillaries) and ii) changes of inflow rate due to arteriole smooth muscle. We showed that a small capillary dilation may lead to an increased RBC heterogeneity in the network. This increased locally the RBC line density and their residence time, in line with previous computational results^21^. Our results also showed, for the first time *in vitro*, the role of increased inflow rate in reducing the RBC heterogeneities in models of microvascular networks. Therefore, both phenomena (local lumen change, variation of inflow rate) were shown to have a significant effect on RBC heterogeneity and may act (possibly simultaneously) to help regulating locally oxygen supply to brain tissue.

## Additional Information

**How to cite this article**: Clavica, F. *et al*. Red blood cell phase separation in symmetric and asymmetric microchannel networks: effect of capillary dilation and inflow velocity. *Sci. Rep.* **6**, 36763; doi: 10.1038/srep36763 (2016).

**Publisher’s note:** Springer Nature remains neutral with regard to jurisdictional claims in published maps and institutional affiliations.

## Supplementary Material

Supplementary Information

## Figures and Tables

**Table 1:** Total hydraulic resistance ratio (R = R_B_/R_B’_) of branches B and B’ in the three models (Fig. 1), calculated using Eq. 1.

	Hydraulic resistance ratio (B/B’)
Symmetric	1
Dilated	1.20
Stretched	0.67
