# Supplementary material for: Red blood cell phase separation in symmetric and asymmetric microchannel networks: effect of capillary dilation and inflow velocity
Source: Sci Rep. 2016 Nov 18;6:36763. doi: 10.1038/srep36763 (PMC5114676; doi:10.1038/srep36763)
Supplement: Supplementary Information [file srep36763-s1.pdf]

# Red blood cell phase separation in symmetric and asymmetric microchannel networks: effect of capillary dilation and inflow velocity

Francesco Clavica[1], Alexandra Homsy[2], Laure Jeandupeux[2], Dominik Obrist[1]

[1]ARTORG Center, University of Bern, Bern, Switzerland

[2]Haute Ecole Arc Ingénierie, HES-SO University of Applied Sciences Western Switzerland, La Chaux-de-Fonds, Switzerland

## Supplementary information

**Table S1:** detailed parameters used for the PTVlab algorithm.

| **PTV settings** | **Particle detection** |
|---|---|
| PTV algorithm = Cross correlation/ Interrogation window. | Detection algorithm = Gaussian mask |
| Interrogation area = 20 pixels | Correlation Threshold = 0.5 |
| Minimum correlation = 0.4 | Sigma = 10 pixels (particle size) |
| Similarity neighbours = 25 % | Intensity threshold = 200 |

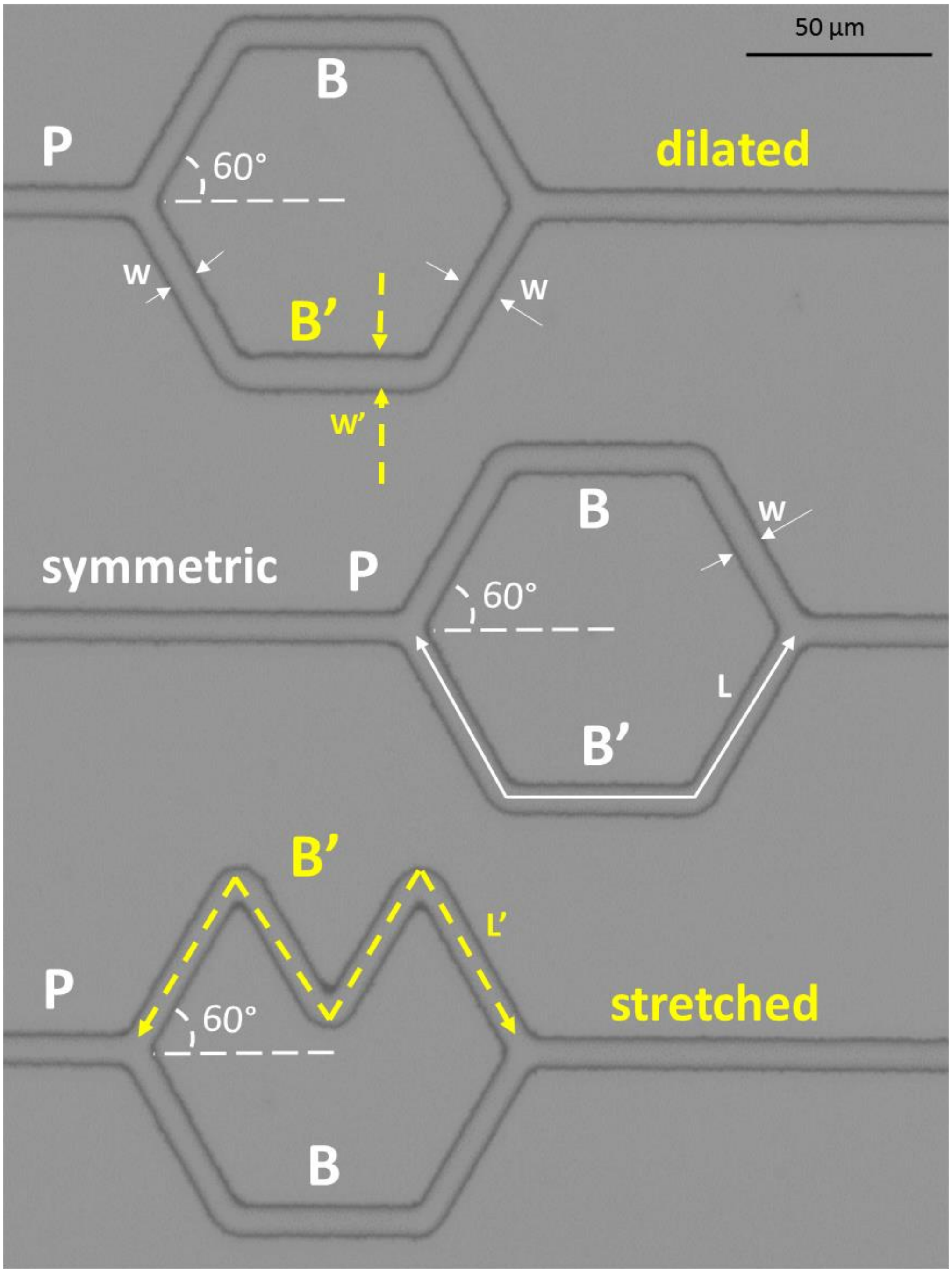


**Figure S1**. **Experimental setup (hexagonal networks).** Microscope image (10x magnification) of hexagonal networks: three different networks were included in the same microdevice, P denotes the parent vessel, B and B' the daughter branches (the bifurcation angle was 60°). B indicates daughter branches characterized by a total length L= 211.38±0.9µm and width W=10.2±0.3µm (N=5). i) Symmetric model (middle) has identical daughter branches B and B' ii) Dilated model (inset, upper) has one daughter branch B and one modified branch B' which has same length as B but different width W'=12.16±0.1µm >W (in the the horizontal segment) iii) Stretched model (inset, lower) has one daughter branch B and one modified branch B' with the same width as B but with different total length L'=259.32±3.1 µm>L (dashed arrows). All models had a common inlet and separate outlets. The average channel height was H=8.1±0.4 µm in all channels. The parent vessel (P) in all the models had a length of 200µm and same width as B branch.

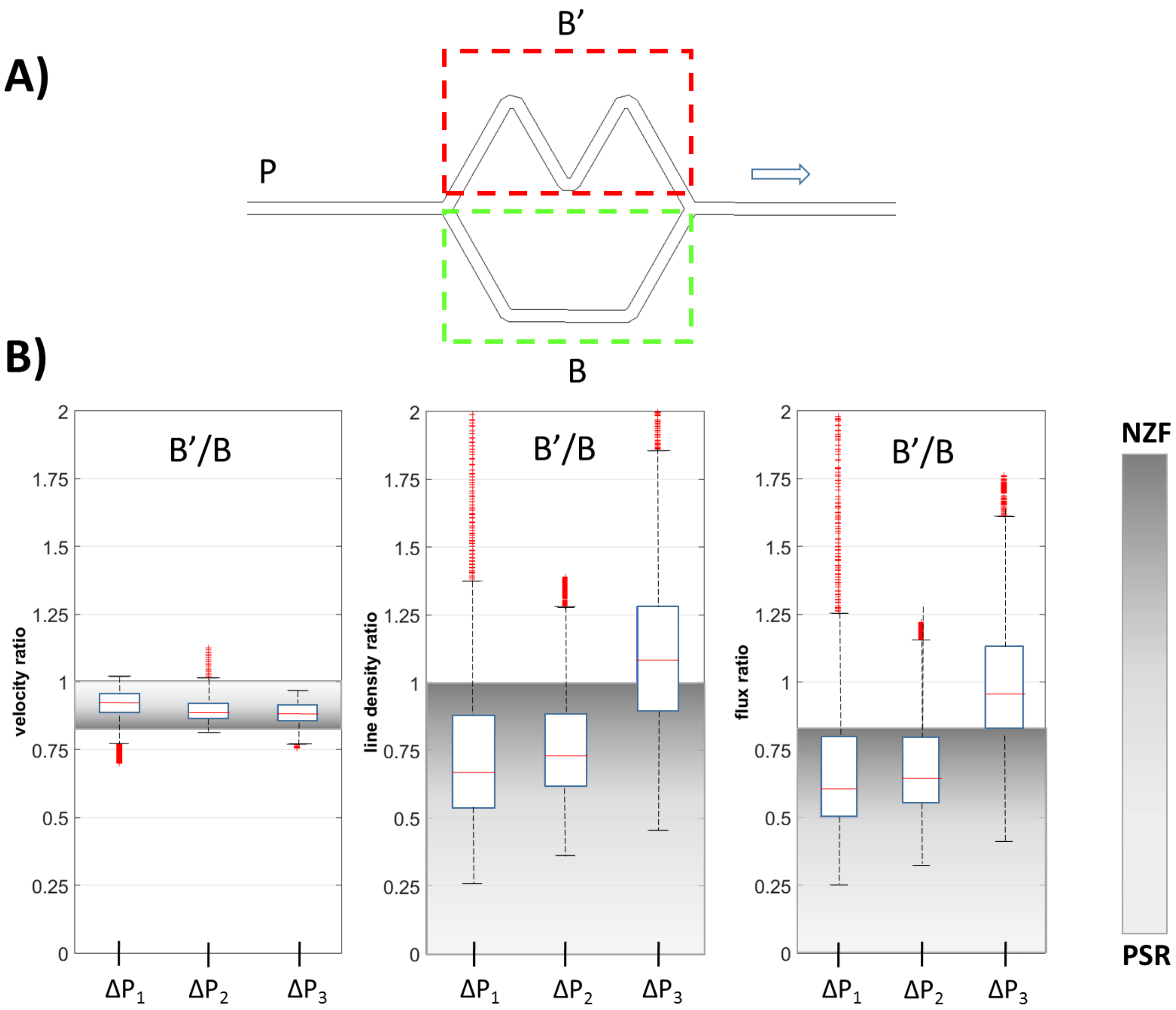


C)

| | $U_p$ (mm/s) | Velocity ratio | Line density ratio | Flux ratio |
|---|---|---|---|---|
| **$\Delta P_1$** | 0.14 | 0.92 | 0.67 | 0.60 |
| **$\Delta P_2$** | 0.38 | 0.88 | 0.73 | 0.64 |
| **$\Delta P_3$** | 0.76 | 0.91 | 1.08 | 0.95 |

**Fig. S2 Effects of inflow rate on phase separation in stretched model (hexagonal).** A) Schematic of the stretched model (hexagonal) with the branch B' longer (ROI: red dashed rectangle) than the B branch (ROI: green dashed rectangle). The arrow indicates the direction of flow. In this experiment three perfusion pressures were used: $\Delta P_1$=2cm$H_2O$, $\Delta P_2$=5cm$H_2O$, $\Delta P_3$=10cm$H_2O$. C) Boxplots of the ratios of RBC velocity, line density and flux of branches B' and B for different perfusion pressures $\Delta P_i$. The shaded areas indicate the range between the no-Zweifach-Fung condition (dark grey, NZF) and perfectly self-regulated situation (light gray, PSR). C) Table of median values of the ratios shown in panel B as function of ΔP. Up indicates the average velocity estimated for the parent vessel.

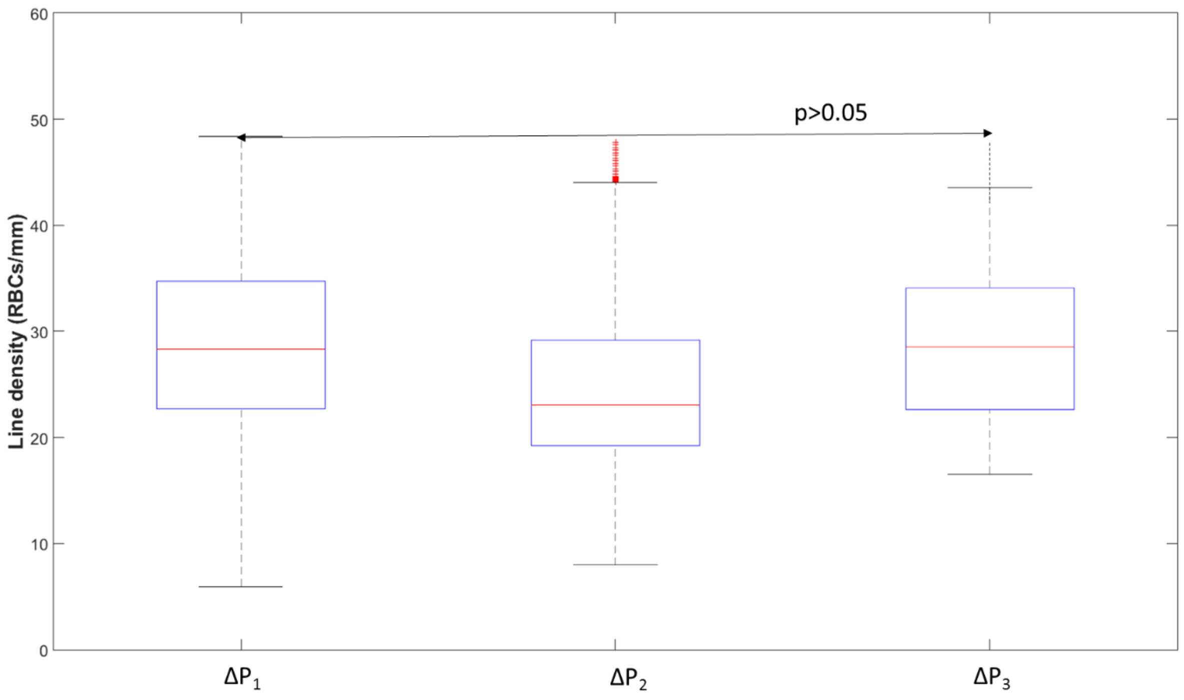


**Fig. S3 Line density vs perfusion pressure in the parent vessel of the stretched mode (T-junction)**
